# Rebalancing power in infectious disease modelling: Toward inclusive and contextual approaches

**DOI:** 10.1371/journal.pgph.0006220

**Published:** 2026-04-03

**Authors:** Justice Moses K. Aheto, Megan Auzenbergs, Matthew J. Ferrari, Allison Portnoy, Chigozie Edson Utazi, Romain Glèlè Kakaï, Ezra Gayawan, James M. Azam, Justice Nonvignon

**Affiliations:** 1 Department of Biostatistics, School of Public Health, College of Health Sciences, University of Ghana, Accra, Ghana; 2 Measles Analytics Hub, Imperial College London, London, United Kingdom; 3 Center for Infectious Disease Dynamics, Pennsylvania State University, University Park, Pennsylvania, United States of America; 4 Department of Global Health, Boston University School of Public Health, Boston, Massachusetts, United States of America; 5 WorldPop, School of Geography and Environmental Science, University of Southampton, Southampton, United Kingdom; 6 Laboratoire de Biomathématiques et d’Estimations Forestières, Faculté des Sciences Agronomiques, Université d’Abomey-Calavi, Cotonou, Benin; 7 Department of Statistics, Federal University of Technology, Akure, Ondo State, Nigeria; 8 Centre for Mathematical Modelling of Infectious Diseases, London School of Hygiene & Tropical Medicine, London, United Kingdom; PLOS: Public Library of Science, UNITED STATES OF AMERICA

## Why now? A critical time for global health equity

Over the past several decades, infectious disease modelling has become a central tool in global health decision‑making, shaping financing decisions, vaccination strategies, and disease control policies [1]; for measles alone, our review identified over 400 modelling studies published since 2000 [2]. However, many of the modelling analyses that have guided these decisions originate in high‑income countries (HICs), even when they intend to inform policy in low- and middle-income countries (LMICs) [3]. With the rapid expansion of Large Language Model (LLM)‑enabled modelling, concerns are intensified about analyses produced without adequate contextual understanding. Models developed at a distance can rely on assumptions that fail to reflect local epidemiology or realities, carrying real‑world consequences for feasibility, equity, and impact.

LLMs, machine learning and other Artificial Intelligence (AI) tools are increasingly being applied in infectious disease modelling, offering rapid data processing and automated model generation—though this is an emerging area, their outputs still require careful validation and contextual interpretation. However, this raises an important question: if anyone can now generate a model using AI, how do we ensure ethics, relevance and local ownership? Recent studies on the utility of LLMs in infectious disease modelling illustrate both promise and limits: Kraemer et al. outline AI’s potential for faster surveillance and forecasting while stressing accountability [4], and Kwok et al. show that AI tools can design models but still require expert validation [5]. Tripathi et al. further emphasise that the benefits of LLMs depend on rigorous validation, transparent processes, and ethical safeguards, stressing that LLMs should complement—not replace—traditional modelling approaches and expertise [6]. Building on this, a roadmap created by Chen et al. highlights that equitable adoption of LLMs in LMICs requires attention to five dimensions—People, Products, Platforms, Processes, and Policies—to avoid reinforcing existing disparities and ensure inclusivity in global health modelling [7].

## Rethinking the architecture of global health financing

As LLMs become more prominent in modelling, it is critical that their adoption does not overshadow local expertise or the human context that ensures models remain ethical, relevant, and grounded in the realities of those most affected. Bann et al. further highlight a major equity challenge: free, open-source LLMs lag far behind pay-for-use, closed-source models, reinforcing past disparities in access to academic resources [8]. Their discussion on “data cleaning” as a “highly manual and time-intensive” task underscores an aspiration for LLMs to reduce unnecessary work, yet these tools cannot replicate the tacit knowledge embedded in the experiences of those who originally collected the data. LLMs can automate mundane aspects of infectious disease modelling such as coding, data cleaning and visualisation, however, their adoption is also hindered by costs. LLM subscription fees are prohibitive for low-resourced researchers from LMICs with current subscription fees ranging between $17 and $200 for LLMs, namely OpenAI’s ChatGPT, Anthropic’s Claude, and Google DeepMind’s Gemini. Inability to adopt these AI tools due to lack of financial support will exacerbate the inequality gap between researchers from LMICs and HICs.

Any integration of LLMs into modelling must respect data sovereignty, protect privacy, and acknowledge local contributors. Financial and environmental costs of training and running LLMs are significant and overlooked; while many platforms currently offer free access, these models rely on subsidies and venture capital that will eventually expire, making pay-for-use subscription models likely and further exacerbating inequities by privileging well-resourced institutions in HICs [7]. This underscores the need for transparent financing strategies and equitable access policies to ensure advances in AI for modelling do not deepen disparities in global health research. At the same time, the growing debate around whether global health initiatives (GHIs) like Gavi and the Global Fund should have “termination dates” highlights the urgency of shifting toward country-led, cost-effective, and accountable models of governance [9]. Modelling communities need fair funding structures that prioritise high-burden countries, ensuring resources are awarded to local modellers.

## The Measles analytics hub: A case study in collaborative modelling

We present a case study of the Measles Analytics Hub (MAH), an initiative built around locally-owned models co-created with in-country experts and global partners to ensure contextual relevance and equity (Supplementary Materials S1 Fig). The MAH, which was established at the end of 2024, exemplifies how modelling can reflect the principles of equity, inclusion, and shared ownership. Funded by the Gates Foundation, the MAH fosters collaboration across its network of members in >50 countries, including high burden countries such as India, Indonesia, Ethiopia, Nigeria, and the Democratic Republic of Congo. Local leadership is embedded in the governance structure rather than being merely symbolic. The Scientific Co-Chair is based in a high-measles-burden country and plays a central role in shaping the research agenda, convening local stakeholders and guiding strategic discussions. MAH members are encouraged to form working groups and actively engage with local stakeholders on programmatically relevant projects such as such as modelling to inform the rollout out of measles Rapid Diagnostic Tests (RDTs), age targeting for future Supplementary Immunisation Activities (SIAs) and subnational risk assessments for measles. The MAH safeguards against pitfalls of LLMs by requiring that any model—including those using AI tools—is co-developed with stakeholders from the country being modelled. This structure ensures that local epidemiological insight remains central and that AI augments, rather than erodes, local ownership. Ongoing discussions within the MAH Global Health Working Group, alongside technical review by the MAH Technical Vetting Committee, provide additional oversight to ensure that AI-assisted modelling remains ethical, contextually grounded, and methodologically robust.

**Fig 1:**
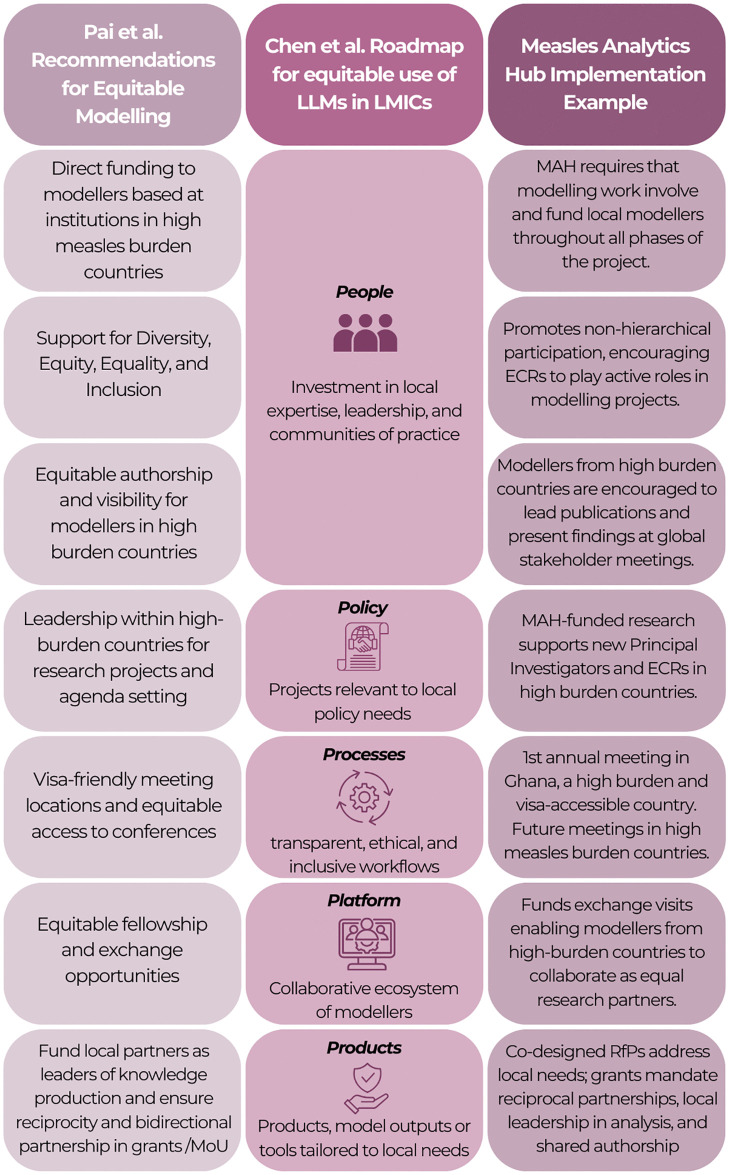
Examples of MAH activities that align with recommendations from Pai et al. (2024) and Chen et al. (2025). MAH = Measles Analytics Hub; MoU = Memorandum of Understanding; ECRs = Early Career Researchers; RfP = Requests for Proposals.

Pai et al. call for “genuine partnerships with people who have lived experience and local expertise” [10], emphasising that sustainable global health leadership must be driven by the Global South with meaningful allyship from the Global North. The MAH demonstrates this in practice and closely aligns with the People–Products–Platforms–Processes–Policies roadmap proposed by Chen et al. Looking ahead, other modelling consortia (i.e., for other diseases) can follow this model by prioritising local leadership, sustained investment in LMIC-based modelling groups, and co‑creation of modelling priorities to ensure that new technologies strengthen, rather than sideline, local expertise. Examples of MAH activities that align with recommendations from Pai et al. (2024) and Chen et al. (2025) are in Fig 1.

## Addressing structural barriers: A call to action

While initiatives like the MAH represent a significant step toward equitable collaboration, structural barriers remain. As AI tools like LLMs make modelling more accessible, the challenge is ensuring that accessibility translates into autonomy, not deeper reliance on HIC networks. Decades of model development and policy engagement have resulted in a dependency on HIC research groups, creating a positive feedback loop that favours established relationships over new voices from high measles burden countries. Global partners often default to HIC institutions because of trust and familiarity, even when technically qualified LMIC experts are available. Breaking this cycle requires more than funding and technical training—it needs deliberate relationship-building, cultural understanding, and mentorship to help LMIC researchers navigate global networks.

Facilitating a successful transition to LMIC leadership is a shared responsibility. HIC researchers must use their access to global partners to create space for LMIC colleagues. This cannot be an LMIC-only effort; it requires joint commitment. Initiatives like the MAH are essential—not only for technical support but also for convening diverse actors and maintaining focus on equitable research partnerships. Funders and global health agencies must recognise these barriers and invest in mechanisms that prioritise trust-building and locally autonomous research outputs.

## Supporting information

S1 Fig Detailed description of how the MAH Secretariat and Modellers and Stakeholders are strategically working together to set working group topics and research priorities. (TIF)
